# αVβ3 Integrin Expression Is Essential for Replication of Mosquito and Tick-Borne Flaviviruses in Murine Fibroblast Cells

**DOI:** 10.3390/v14010018

**Published:** 2021-12-23

**Authors:** Vinicius Pinho dos Reis, Markus Keller, Katja Schmidt, Rainer Günter Ulrich, Martin Hermann Groschup

**Affiliations:** 1 Institute of Novel and Emerging Infectious Diseases, Friedrich-Loeffler-Institut, Federal Research Institute for Animal Health, Südufer 10, 17493 Greifswald-Insel Riems, Germany; vinicius.pinhodosreis@uni-marburg.de (V.P.d.R.); Markus.Keller@fli.de (M.K.); rainer.ulrich@fli.de (R.G.U.); 2 Institute for Virology, Philipps University Marburg, Hans-Meerwein-Straße 2, 35043 Marburg, Germany; 3 Microbiological Diagnostics, German Cancer Research Center, Im Neuenheimer Feld 280, 69120 Heidelberg, Germany; katja.schmidt@dkfz-heidelberg.de; 4 Deutsches Zentrum für Infektionsforschung(DZIF), Partner Site Hamburg-Lübeck-Borstel-Riems, Südufer 10, 17493 Greifswald-Insel Riems, Germany

**Keywords:** integrins, flavivirus, host cell factors, virus binding, virus internalization, virus replication

## Abstract

The *Flavivirus* genus includes a number of important viruses that are pathogenic to humans and animals and are responsible for outbreaks across the globe. Integrins, a family of heterodimeric transmembrane molecules expressed in all nucleated cells mediate critical functions of cell physiology and cell cycle. Integrins were previously postulated to be involved in flavivirus entry and to modulate flavivirus replication efficiency. In the present study, mouse embryonic fibroblasts (MEF), lacking the expression of αVβ3 integrin (MEF-αVβ3^−/−^), were infected with four different flaviviruses, namely yellow fever virus (YFV), West Nile virus (WNV), Usutu virus (USUV) and Langat virus (LGTV). The effects of the αVβ3 integrin absence in double-knockout MEF-αVβ3^−/−^ on flavivirus binding, internalization and replication were compared to the respective wild-type cells. Binding to the cell surface for all four flaviviruses was not affected by the ablation of αVβ3 integrin, whereas internalization of USUV and WNV was slightly affected by the loss of αVβ3 integrin expression. Most interestingly, the deletion of αVβ3 integrin strongly impaired replication of all flaviviruses with a reduction of up to 99% on virus yields and a strong reduction on flavivirus anti-genome RNA synthesis. In conclusion, our results demonstrate that αVβ3 integrin expression in flavivirus-susceptible cell lines enhances the flavivirus replication.

## 1. Introduction

Zoonotic flaviviruses (family *Flaviviridae*; genus *Flavivirus*) are able to infect a broad diversity of hosts such as equines, birds, non-human primates and humans (reviewed in [1,2]). Among these, viruses as dengue virus (DENV), yellow fever virus (YFV), West Nile virus (WNV), Usutu virus (USUV), Zika virus (ZIKV) and tick-borne encephalitis virus (TBEV) are responsible for disease outbreaks in tropical and subtropical regions [2]. In most cases, flavivirus infection leads to subclinical manifestations or self-limited *flu-like* symptoms. Rare but severe neurological manifestations like encephalitis, Guillain Barré syndrome or acute flaccid paralysis are also reported upon flavivirus infection in endemic areas [3,4]. Flaviviruses are enveloped with a single-stranded RNA genome of positive polarity (+ssRNA). The genomic RNA encodes for a polyprotein that is cleaved into 10 proteins: three structural and seven non-structural proteins [5].

Upon attachment to cellular receptor(s), virions are internalized mainly through clathrin-mediated endocytosis [6]. Within the endosomes, the low pH environment triggered by lysosome fusion causes conformational changes in the envelope protein resulting in viral membrane fusion and release of the viral genome into the cytoplasm, initiating the replication cycle [5,7]. During flavivirus RNA replication, an intermediate double-stranded RNA (dsRNA) form is synthetized, which is later unwound by a viral helicase. The antigenome RNA serves as template for the synthesis of new flavivirus genomic RNA (+ssRNA) [5]. Their exceptional ability to infect a broad diversity of hosts raises speculations that flaviviruses use a common-shared receptor(s) to infect the host cell [8]. In the last few years, several molecules have been characterized to be involved in the early steps of flavivirus infection such as heat shock proteins, TIM and TAM receptors, heparan sulfate and Dendritic Cell-Specific Intercellular Grabbing Non-integrin molecules (DC-SIGN) and integrins (reviewed in [8,9,10]).

Integrins are a class of cell adhesion receptors and are expressed essentially in all nucleated cells from nematodes to mammals [11]. They are heterodimeric transmembrane molecules composed of two non-covalently linked subunits: one alpha (α) and one beta (β) integrin subunit [12]. Up to now, 18 α integrin subunits and 8 β integrin subunits have been identified and characterized. These α and β integrin subunits combine to each other to form 24 different integrin heterodimers [13]. The main function of integrin molecules is to project cell adhesion via extracellular matrix interaction. These interactions promote intracellular signaling that regulates important cellular functions like cell motility, survival, differentiation and division [14,15]. Integrins have been postulated as cellular receptors for several viruses, like foot and mouth disease virus, human adenoviruses, hantaviruses and human echovirus [16,17,18,19,20,21,22]. In addition to that, several other viruses exploit the integrin machinery to promote attachment and internalization (i.e., Coxsackievirus and Ebola virus and some members of the *Herpesviridae* family) [22,23,24,25,26]. Flavivirus and integrin interactions were first reported by Chu et al., who demonstrated that αVβ3 integrin mediated WNV binding to and entry into CS-1 human melanoma cells. Furthermore, functional blocking of αVβ3 integrin as well as αVβ3 integrin gene silencing resulted in inhibition of WNV entry and infection leading to the conclusion that αVβ3 integrin is a putative receptor for WNV [27,28]. In contrast, Medigeshi et al. demonstrated later that WNV binding to and entry into the host cells are completely independent of αVβ3 integrin [29]. More recently, αVβ3 and αVβ5 integrin heterodimers were shown to be involved in Japanese encephalitis virus (JEV) and ZIKV infection and internalization, respectively, in human and mouse cell lines [30,31].

We previously reported that WNV binding and entry is independent of integrins for all four WNV strains tested in a mouse cell model lacking the expression of β3 and β1 integrin subunits. However, integrin expression substantially enhanced WNV replication raising speculations that integrins might represent an important host cell factor for WNV infection and replication [32]. In the present study, we investigated the role of αVβ3 integrin in infection with WNV and three other flaviviruses using a mouse embryonic fibroblast (MEF) model lacking the expression of αVβ3 integrin. Our results demonstrated that the loss of αVβ3 integrin expression did neither impair flavivirus binding to the cell surface nor affected cell susceptibility. Most importantly, ablation of αVβ3 integrin substantially affected flavivirus RNA replication suggesting that αVβ3 integrin represents an essential host cell factor, which promotes effective flavivirus replication.

## 2. Materials and Methods

### 2.1. Cell Lines

MEFs harboring a genomic ablation of the αV and β3 integrin subunit genes (MEF-αVβ3^−/−^) and its respective wild-type cells (MEF-WT) were cultivated in Dulbecco’s Modified Eagle’s Medium (DMEM, ThermoFisher, Waltham, MA, USA) supplemented with 10% Fetal Bovine Serum (FBS, ThermoFisher, Waltham, MA, USA). The production of MEFs harboring genomic ablations of integrin genes is described elsewhere [32]. CHO-K1 cells (CHO-K1, ATCC n° CCL61) and Vero cells (clone 76, ATCC n° CRL-1587) were obtained from the Collection of Cell Lines from the Friedrich-Loeffler-Institut (FLI, Greifswald-Insel Riems, Germany). CHO-K1 and Vero 76 cells were cultivated in Minimum Essential Medium (MEM, ThermoFisher, Waltham, MA, USA) supplemented with antibiotics and 5% of FBS. All cells were maintained at 37 °C and 5% CO_2_.

### 2.2. Viruses

Yellow fever virus strain 17D (YFV-17D [33]), Usutu virus strain Germany (USUV, [34]), West Nile virus vaccine strain (PrevNile, [35]) and Langat virus strain TP-21 (LGTV, [36]) were kindly provided by Dr. Ute Ziegler (INNT-FLI, Greifswald-Insel Riems). Viruses were inoculated into semi-confluent monolayers of Vero 76 cells for 5–7 days. Supernatants from infected cells were harvested, centrifuged at 5000 revolutions per minute (rpm) for 10 min, aliquoted and stored at −80 °C until use.

### 2.3. Determination of Virus Titers

Viral stock titers were determined by plaque assay as described elsewhere [37]. Briefly, virus stocks were ten-fold serially diluted in MEM supplemented with antibiotics but without FBS. Dilutions with a final volume of 1 mL were added to each well in duplicates. Plates were incubated for 1 h at 37 °C with 5% CO_2_ and agitation every 15 min to ensure efficient virus adsorption. After 1 h, inocula were removed and 3 mL of overlay medium (MEM with 2% FBS, antibiotics and 1.8% bacteriological-grade agarose) added and incubated at 37 °C with 5% CO_2_ for 7 days. At day 7 post inoculation, plates were fixed with 10% buffered formalin and stained with 1% crystal violet solution. Plaques were counted and the titer determined as follows: (n° of plaques × inoculum volume)/inverse dilution = titer in Plaque Forming Units/mL (PFU/mL)). Tissue culture infectivity dose 50 (TCID_50_) was used to estimate the endpoint titers in infection experiments. For this, Vero 76 cells were seeded into 96-well plates at a concentration of 10^5^ cells per well 24 h prior to virus inoculation. One hundred microliter of supernatants from infected cells were added to the first column and ten-fold serially diluted in MEM supplemented with antibiotics but without FBS. Plates were then incubated at 37 °C with 5% CO_2_ for 1 h for virus adsorption. Thereafter, inocula were removed, plates washed once with 1× phosphate-buffered saline (PBS) which was replaced with fresh MEM supplemented with 2% FBS and antibiotics. Plates were then incubated for 7 days at 37 °C with 5% CO_2_. On the 7th day, supernatants were removed, and cell monolayers were fixed with 10% buffered formalin for 1 h, followed by washing to remove cell debris and eventually stained with 1% crystal violet solution for 1 h. Plates were extensively washed and dried overnight. Spearman and Kärber calculation was applied to determine TCID_50_ as described elsewhere [38,39].

### 2.4. Antibodies

Hamster anti-mouse CD61 (integrin β3) clone HM-β 3.1, rat anti-mouse CD51 (integrin αV) clone RMV-7 and hamster anti-mouse CD29 (integrin β1) clone HMβ1–1 were purchased from Biolegend (San Diego, CA, USA). Biotin-labeled hamster anti-mouse CD61 (integrin β3) clone HM-β 3.1 and rat anti-mouse CD51 (integrin αV) clone RMV-7 were used for cell sorting. Anti-hamster immunoglobulin G (IgG) labeled with Alexa 488, anti-rat IgG-Alexa 647 and anti-rat IgG-Cyanine 3 (Cy3) were purchased from Jacksons Immunoresearch (Cambridge, UK). Rat and Armenian hamster IgG isotype controls were purchased from Biolegend (San Diego, CA, USA).

### 2.5. Flow Cytometry Analysis

Flow cytometry analysis was conducted to estimate the levels of integrin expression on the cell surface. Briefly, cells were detached from cell culture flasks and passed through a 40 μm cell strainer (BD Bioscience, San Jose, CA, USA). Cells were counted in hematocytometer and a cell concentration adjusted to 1 × 10^6^ cells per tube. Cells were incubated on ice for 30 min, centrifuged and incubated with integrin-subunit specific antibody for 1 h at 4 °C. After this incubation, cells were washed twice with cold 1× PBS and centrifuged at 2000 rpm for 5 min. Secondary antibodies labelled with fluorescent dyes were added and incubated for an additional 1 h at 4 °C. Subsequently, cells were washed twice with cold 1× PBS and centrifuged at 2000 rpm for 5 min. Cell pellets were resuspended in cold 1× PBS before integrin expression was measured by BD FACSCanto II (BD Bioscience, San Jose, CA, USA) flow cytometer and acquired with BD FACSDiva Software (BD Bioscience, San Jose, CA, USA). Flow cytometry data were analyzed using Flowing software version 2 (Turku Centre for Biotechnology, Turku, Finland).

### 2.6. Cloning, Cell Transfection and Cell Sorting

CHO-K1 cells were transfected with vectors encoding either the mouse αV or the mouse β3 integrin subunits (accession numbers: KP296148.1; NM016780.2). The αV and β3 integrin subunit encoding sequences were inserted into pcDNA3.1-based plasmids (ThermoFisher, Waltham, MA, USA) between the BamHI and NotI restriction sites (pcDNA 3.1 Zeo-β3 and pcDNA 3.1 Hygro-αV). CHO-K1 transfection was performed using Lipofectamine 3000 (ThermoFisher, Waltham, MA, USA) at a DNA/Lipofectamine ratio of 1:3. Briefly, 1 × 10^6^ cells were seeded in 12 well-plates 12–16 h prior transfection. Twenty-four hours post-transfection, medium was changed and cells incubated for additional 24 h. Forty-eight hours post-transfection, cells were split and set under antibiotic selection markers as indicated: Hygromycin (ThermoFisher, Waltham, MA, USA) 100 μg/mL and Zeocin (Invivogen, San Diego, CA, USA) 500 μg/mL. After antibiotic selection, cells were sorted by positive selection using MACS Microbeads magnetic cell sorting system (Miltenyi Biotec, Gladbach, Germany) following manufacturer’s instructions. Shortly, cells were detached and resuspended in sterile 1× PBS containing 0.5 g sodium azide/L and 1% bovine serum albumin (BSA) fraction V (Sigma-Aldrich, St. Louis, MO, USA) and incubated with biotin-labeled hamster anti-mouse CD61 (integrin β3) and rat anti-mouse CD51 (integrin αV) antibodies for 2 h at 4 °C with constant rotation. After incubation, cells were washed twice with cold 1× PBS and incubated with anti-biotin MACS Microbeads (Miltenyi Biotec, Gladbach, Germany) for 30 min at 4 °C with constant rotation. Subsequently, cells were washed twice with cold 1× PBS and loaded onto magnetic MS-Columns (Miltenyi Biotec, Gladbach, Germany). The flow-through containing the integrin negative cells was discarded and after extensive washes the integrin expressing cells were eluted, centrifuged and cultivated in medium containing zeocin or hygromycin selection antibiotics as mentioned above.

### 2.7. Indirect Immunofluorescence Assay

Cells were grown on glass cover slips 12–16 h prior to immunostaining. Thereafter, cells were fixed with 3% paraformaldehyde (PFA, Carl Roth, Karlsruhe, Germany) for 15 min, followed by incubation with 50 mM ammonium chloride (Carl Roth, Karlsruhe, Germany) for 30 min. After this, cells were permeabilized with 0.5% TritonX-100 (Carl Roth, Karlsruhe, Germany), washed twice with 1× PBS and blocked with 0.5% skimmed milk (Carl Roth, Karlsruhe, Germany) diluted in 1× PBS. Antibodies were diluted in blocking buffer and cells were incubated with integrin-specific antibodies for 1 h at room temperature followed by three washes with 1× PBS and subsequent incubation with secondary Alexa 488-labeled anti-hamster IgG and Cy3-labeled anti-rat IgG antibodies for an additional 1 h at 4 °C. For nuclei staining, the glass cover slips were quickly rinsed with 2 mg/mL of 4’,6-diamidino-2-phenylindole (DAPI) solution (Sigma-Aldrich, St. Louis, MO, USA) diluted at 1:5000 in 1× PBS followed by a final wash with 1× PBS plus a quick rinse in distilled water. Finally, coverslips were dried and fixed upside down on microscopy slides with VectaShield anti-fade mounting medium (Vector labs, San Francisco, CA, USA). Cells were visualized in the laser-scanning confocal microscope Leica DMI600 CS using the LAS AF Leica Application Suite software (Leica, Wetzlar, Germany). Images were post-treated with ImageJ software (National Institutes of Health, Bethesda, MD, USA).

### 2.8. Cell Viability Assay

Determination of cell viability was performed using the CellTiter 96^®^ AQueous One Solution Cell Proliferation Assay (Promega, Madison, WI, USA) following instructions of the manufacturer. Briefly, cells were detached and resuspended in DMEM containing 10% FBS. Total cell number was determined and cells were seeded in different concentrations ranging from 10^3^ to 10^6^ cells per well in duplicate into 96-well plates to a final volume of 100 μL. Subsequently, 20 μL of 3-(4,5-dimethylthiazol-2-yl)-5-(3-carboxymethoxyphenyl)-2-(4-sulfophenyl)-2H-tetrazolium (MTS) reagent was added to each well, plates were gently agitated and incubated at 37 °C with 5% CO_2_ atmosphere under light protection for 4 h. After 4 h, absorbances were measured at 490 nm using an ELISA plate reader (TECAN, Männedorf, Switzerland). Background wells (only MTS reagent) as well as blank wells (no reagent) were added as controls. Absorbances were plotted by the mean value of respective cell amount after background absorbance subtraction.

### 2.9. Cell Adhesion Assay

Cell adhesion assays were performed as described elsewhere with modifications [40]. Briefly, Maxisorp ELISA plates (ThermoFisher, Waltham, MA, USA) were coated overnight at 4 °C with 1 μg/mL of recombinant mouse vitronectin (Sino Biological, Beijing, China) or BSA in carbonate buffer (pH 8.0). The next day, plates were washed once with 1× PBS and blocked with 2% BSA solution for 1 h at 37 °C. Cell monolayers were detached from the cell culture flasks using 5mM ethylenediaminetetraacetic acid (EDTA, Carl Roth, Karlsruhe, Germany), the total cell number determined and added to the wells at a final concentration of 1 × 10^5^ cells per well in serum free medium supplemented with 0.1% BSA. Cells were incubated for 45 min at 37 °C to allow adhesion to vitronectin. Vero 76 cells were used as attachment controls for the assay. After adhesion, plates were gently washed to remove unbound cells and plates were fixed with 3% PFA for 1 h at room temperature. Adherent cells were stained for 1 h with 1% crystal violet prepared in 20% methanol (Carl Roth, Karlsruhe, Germany). After extensive washing, plates were dried and dye was extracted from the cells using 50% ethanol (Carl Roth, Karlsruhe, Germany) in 50 mM sodium citrate buffer (pH 4.5, Carl Roth, Karlsruhe, Germany). Optical densities (OD) were measured at 550 nm using an ELISA plate reader (TECAN, Männedorf, Switzerland).

### 2.10. Virus Infection Experiments

Multi-cycle replication kinetics were performed in 24-well plates. For this, cells were seeded at concentration of 1 × 10^4^ cells per well at 12 h prior to virus inoculation and cultured with DMEM supplemented with 2% FBS. Then, cells were washed once with 1× PBS and inoculated with different flaviviruses at a multiplicity of infection (MOI) of 0.1 for 1 h at 37 °C. Inocula were removed and cells washed twice with 1× PBS before 1 mL of DMEM supplemented with 2% FBS was added to each well. Supernatants were harvested at 24, 48, 72 and 96 h post inoculation and virus titers were determined by TCID_50_ in Vero 76 cells as described above.

For the binding assay, cells were seeded on 12-well plates with DMEM supplemented with 2% FBS at a concentration of 1 × 10^5^ cells per well, at 12 h prior virus inoculation. On the next day, medium was replaced by serum free DMEM and cells were pre-incubated at 4 °C for 30 min prior to virus inoculation. After this, plates were placed on ice and cells inoculated with the flaviviruses at a MOI of 10. Cells were incubated for 1 h on ice with agitation every 15 min. Thereafter, the inoculum was removed and the wells were washed four times with cold 1× PBS to remove unbound virus particles. Cell monolayers were resuspended in RLT buffer (Qiagen, Hilden, Germany) and stored at −80 °C.

For the internalization assay, cells were seeded as described above. After pre-incubation of cells at 4 °C for 30 min, virus inoculation and incubation on ice, virus inocula were removed and cells were washed three times with cold 1× PBS and shifted to 37 °C for 40 min to allow virus internalization. Thereafter, medium was removed and cell monolayers were washed once with cold 1× PBS and treated with acidic glycine (pH 2.5, Carl Roth, Karlsruhe, Germany) for 2 min to inactivate non-internalized virions as described elsewhere [41,42,43] followed by 2 washes with 1× PBS. Cell monolayers were resuspended in RLT buffer (Qiagen, Hilden, Germany) and stored at −80 °C.

For the replication assay, cells were seeded as described above. Cells were inoculated with flaviviruses at a MOI of 10 and incubated at 37 °C for virus adsorption for 1 h. After this period, inocula were removed and monolayers were washed four times with 1× PBS. Finally, cells were cultivated with DMEM containing 2% FBS and incubated at 37 °C with 5% CO_2_. Supernatants and monolayers were harvested 48 h after inoculation and stored at −80 °C until use.

### 2.11. Binding Inhibition Assay

For the binding inhibition assay, cells were seeded on 24-well plates with DMEM supplemented with 2% FBS at a concentration of 1 × 10^5^ cells per well, 12–16 h prior to virus inoculation. Medium was replaced by serum free DMEM medium and cells were pre-incubated at 4 °C for 15 min prior to treatment with integrin ligands. Type I collagen (0–500 μg/mL-Sigma-Aldrich, St. Louis, MO, USA), synthetic Arg-Gly-Asp (RGD motif) tripeptide (0–250 μg/mL-Sigma-Aldrich, St. Louis, MO, USA) and recombinant mouse vitronectin (0–50 μg/mL-Sino Biological, Beijing, China) were added into the wells with serum free DMEM supplemented with 1 mM MnCl_2_ (Carl-Roth, Karlsruhe, Germany) and 1 mM MgCl_2_ (Carl-Roth, Karlsruhe, Germany) and incubated at 4 °C for 30 min to allow ligand binding. After this, plates were placed on ice and washed twice, and cells were inoculated with flaviviruses at a MOI of 10. Cells were incubated for 1 h on ice with constant agitation every 15 min. After this period, the inoculum was removed and the wells were washed four times with cold 1× PBS to remove unbound virus particles. Cell monolayers were resuspended in RLT buffer (Qiagen, Hilden, Germany) and stored at −80 °C.

### 2.12. RNA Isolation and RT-qPCR

Viral RNA from cell supernatants was isolated using the Qiamp Viral RNA Mini Kit (Qiagen, Hilden, Germany) following manufacturer instructions. Total RNA from infected and uninfected cells was isolated using the RNeasy Mini Kit (Qiagen, Hilden, Germany) following manufacturer instructions. RNA samples were stored at −80 °C until use. To quantify the viral genomic RNA, reverse transcription quantitative polymerase chain reaction (RT-qPCR) was performed using the QuantiTect Probe RT-PCR kit (Qiagen, Hilden, Germany). For quantification of viral genome, a standard curve containing serial dilutions of a synthetic RNA harboring the primer/probe binding sites of each primer set used was included in every RT-qPCR run.

The thermal cycling conditions used for RT-qPCR were as follows: 50 °C for 30 min followed by 95 °C for 15 min for reverse transcription and 45 cycles at 95 °C for 15 s, 55 °C for 30 s, and 72 °C for 15 s for qPCR. For the detection of flavivirus antigenomic RNA from infected monolayers, total RNA was isolated as described above and cDNA was synthetized using the respective flavivirus specific forward primers described in Table S1. The cDNA synthesis was performed using SuperScript III reverse transcriptase (ThermoFisher, Waltham, MA, USA) as follows: 25 °C for 10 min, 50 °C for 60 min and 85 °C for 15 min. Levels of flavivirus antigenome RNA were normalized to beta-actin as housekeeping gene and calculated using the 2^−ΔΔCt^ method [44]. The cDNA synthesis was performed on Biometra T3 Thermal Cycler (Analytik Jena, Jena, Germany) and RT-qPCR reactions were performed on CFX96 Real-Time PCR System (Bio-Rad, Feldkirchen, Germany). Primer and probe sequences are listed in Supplementary Table S1.

### 2.13. Data and Statistical Analysis

Primary data were analyzed using Microsoft Excel and graphics were designed using GraphPad Prism version 8.4 (San Diego, CA, USA). Statistical analyses were performed using GraphPad Prism version 8.4. The non-parametric Mann-Whitney-U test was applied to evaluate data from the binding experiments. The parametric Student’s *t*-test was applied to evaluate data from replication and internalization assays. One-Way ANOVA with Bonferroni’s correction was applied for the replication experiments with the CHO-K1 cells. Statistical significance is represented as: (*) = *p* ≤ 0.05, (**) = *p* ≤ 0.01, (***) = *p* ≤ 0.001 and (****) = *p* ≤ 0.0001. Non-significant: (ns) = *p* > 0.05.

## 3. Results

### 3.1. Characterization of Integrin-Deficient Cell Lines

**Figure 1:**
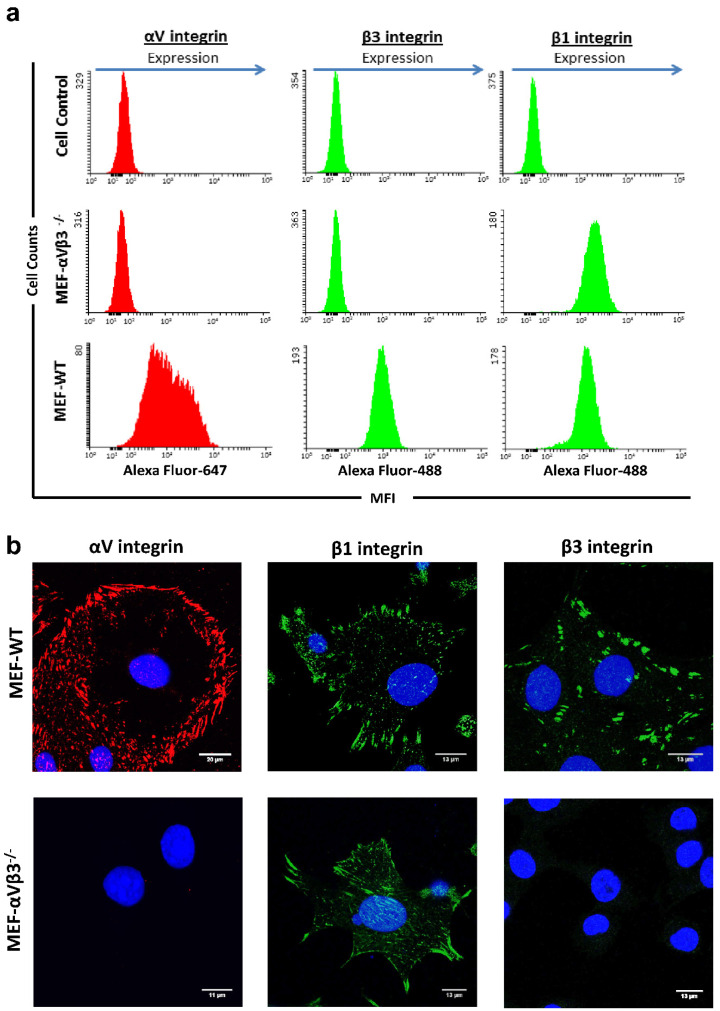
Integrin expression patterns in MEF-WT and MEF-αVβ3^−/−^. Cells were analyzed by flow cytometry (**a**) and laser scanning confocal microscopy (**b**). In (**a**), cells were incubated with anti-integrin subunit-specific antibodies followed by incubation with secondary antibodies labelled with Alexa 647 (red histograms) or Alexa 488 (green histograms). Cell controls correspond to untreated cells. Histograms represent the mean fluorescence intensity (MFI; *x*-axis) and the cell counts (*y*-axis). Total cell counts: 1 × 10^4^. In (**b**) cells were cultivated on coverslips and stained with integrin subunit-specific antibodies followed by incubation with secondary antibodies labelled with Alexa 488 (green) or Cy3 (red). Nuclei were stained with DAPI. Laser wavelengths used for imaging: 405 nm (DAPI), 450–470 nm (Alexa 488), 540–570 nm (Cy3). Scale bar: 20 μm (αV integrin) and 13 μm (β1 and β3 integrins).

MEFs deficient for αVβ3 (MEF-αVβ3^−/−^), as well as the respective wild-type (MEF-WT) cell lines were characterized for their integrin expression profile by flow cytometry and confocal laser scanning microscopy. First, flow cytometry analysis was performed to confirm loss of αVβ3 integrin expression in the integrin deficient cell line as well as to measure the level of integrin expression in the MEF-WT. The MEF-WT expressed high amounts of αV and β3 integrin subunits, whereas the MEF-αVβ3^−/−^ showed complete absence of both, the αV and β3 integrin subunits (Figure 1a). We also measured the levels of β1 integrin subunit, which is widely expressed in murine fibroblasts [32,45]. The β1 integrin subunit could be detected on the cell surface of the MEF-WT as well as MEF-αVβ3^−/−^ indicating that other integrin heterodimers are present on the cell surface of MEF-αVβ3^−/−^ (Figure 1a). Expression levels of αV, β1 and β3 integrin subunits reached more than 99% in the MEF-WT, contrary to the MEF-αVβ3^−/−^, which demonstrated total absence of αV and β3 integrin subunit expression whereas high amounts of β1 integrin subunit were still expressed (>99%). Ablation of αVβ3 in the MEF-αVβ3^−/−^ had no influence on cell viability when compared to the MEF-WT (Figure S1a). Chinese hamster ovary (CHO) cells transfected with either the mouse αV (CHO-αV^+/+^) or the mouse β3 (CHO-β3^+/+^) integrin subunit expressed high amounts of the respective integrin subunit (Figure S2a). In order to confirm these findings and to determine the integrin subcellular localization, confocal laser scanning microscopy was applied. As demonstrated in Figure 1b, if expressed, integrins are globally distributed over the cell membrane in both MEF-WT and MEF-αVβ3^−/−^ (Figure 1b). In accordance with the flow cytometry analysis, the MEF-WT expressed significant amounts of αV, β1 and β3 integrin subunits, whereas MEF-αVβ3^−/−^ showed to solely express the β1 integrin subunit (Figure 1b). The integrin subcellular localization as well as their expression patterns are in accordance with what was previously described [32,46]. Similarly, high amounts of both αV and β3 integrin subunit in the CHO-αV^+/+^ and CHO-β3^+/+^ cells were visualized by confocal microscopy as shown in Figure S2b.

### 3.2. Integrins Are Not Involved in Flavivirus Binding to the Host Cell

**Figure 2:**
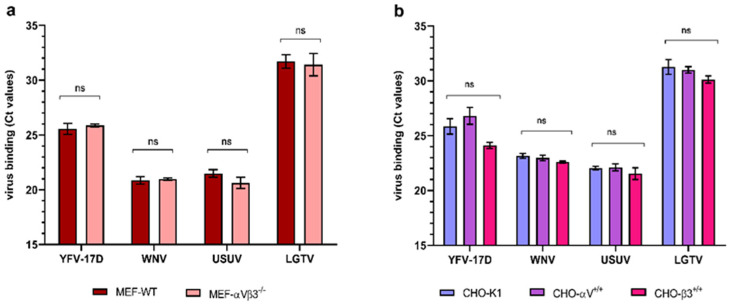
Flavivirus binding to the cell surface of (**a**) MEF-WT and MEF-αVβ3^−/−^ and (**b**) CHO-K1, CHO-αV^+/+^ and CHO-β3^+/+^. Cells were infected with YFV-17D, WNV, USUV or LGTV at a MOI of 10, placed on ice to allow virus binding to the cell surface but no internalization. After 1 h, cells were extensively washed, monolayers harvested and the amount of virus bound to the cell surface was determined by RT-qPCR. Virus binding values are expressed in cycle threshold (Ct) values. Three independent experiments were performed in triplicate each. Bars represent mean Ct values and error bars represent the standard deviation (means ± standard deviation). Statistical analysis: Mann–Whitney test; ns: not significant (*p* > 0.05).

One of the main integrin functions is to mediate cell adhesion through close interaction with the extracellular matrix [12,14]. Previous reports have shown that several viruses use integrins as an attachment factor to trigger virus internalization pathways [17,23,47]. To investigate whether αVβ3 integrin is involved in flavivirus binding to the host cells, MEF-αVβ3^−/−^ and MEF-WT cells were inoculated with different flaviviruses. Cell monolayers were initially pre-incubated at 4 °C to arrest cell membrane movement, and consequently virus internalization, followed by incubation on ice and inoculation with different flaviviruses. As shown in Figure 2a, the absence of αVβ3 integrin did not impair flavivirus binding to the cell surface of MEF-αVβ3^−/−^ cells as measured by RT-qPCR. In fact, flavivirus binding to both MEF-αVβ3^−/−^ and MEF-WT were at a similar level to that observed for Vero 76 cells (Figure S3). Similarly, ectopic expression of murine αV or β3 integrin subunits in CHO cells, a cell line which does not express αV or β3 integrin subunits on the cell surface, did not enhance flavivirus binding to the cell surface (Figure 2b). Since the deletion of the αV integrin subunit impairs the formation of αV-containing integrin he-terodimers other than αVβ3 (i.e., αVβ1, αVβ5, αVβ6, αVβ8) at the cell surface, other integrin heterodimers might still be present in the MEF-αVβ3^−/−^. To investigate whether these integrin heterodimers other than αVβ3 integrin are involved in flavivirus binding, MEF-WT as well as MEF-αVβ3^−/−^ were treated with different commonly used integrin ligands, such as: vitronectin, type I collagen and a synthetic RGD tripeptide motif. For this purpose, cells were incubated at 4 °C to allow ligand binding and subsequently infected with different flaviviruses. As denoted in Figure S4, cell treatment with different integrin ligands had no impact on flavivirus binding to the cell surface of MEFs, reinforcing that integrins play no role in flavivirus binding to the MEFs. Together, these results demonstrate that αVβ3 integrin and other integrins that interact with synthetic integrin ligands are not required for flavivirus binding to the cell surface of MEF.

### 3.3. Ablation of αVβ3 Integrin Expression Does Not Hinder Flavivirus Infection

**Figure 3:**
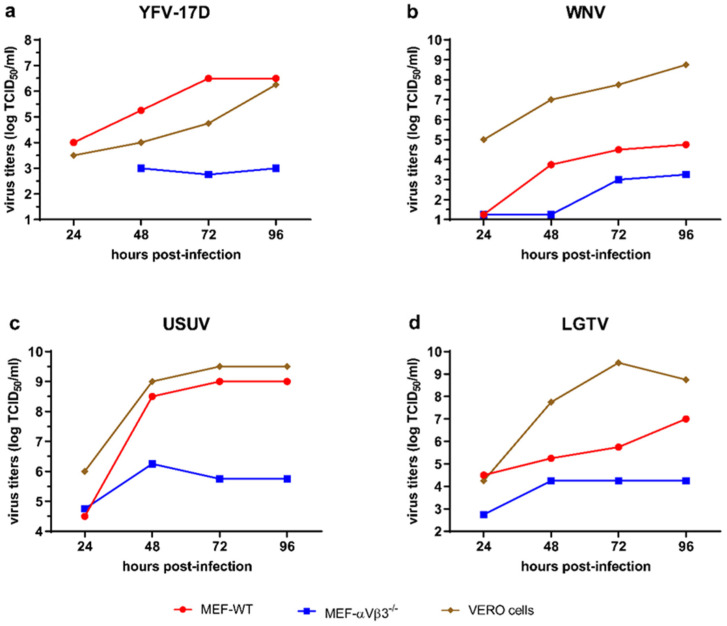
Replication kinetics of (**a**) YFV-17D, (**b**) WNV, (**c**) USUV and (**d**) LGTV in MEF-WT, MEF-αVβ3^−/−^ and Vero 76 cells. Cells were inoculated with these flaviviruses at a MOI of 0.1 and supernatants were harvested at 24, 48, 72 and 96 h post inoculation. Values represent virus titers determined by TCID_50_ in Vero 76 cells over a period of 4 days post-infection.

In order to verify whether genomic deletion of αVβ3 integrin in MEFs renders cells resistant to flavivirus infection, we performed a multi-step virus replication kinetics. MEF-WT as well as MEF-αVβ3^−/−^ were inoculated with YFV-17D, WNV, USUV and LGTV at a MOI of 0.1 to assess the flaviviruses ability to infect and replicate in these cells. Supernatants were harvested every 24 h until 4 days post-inoculation, and viral titers were determined by TCID_50_ assay. Highest titers were achieved for WNV, LGTV and USUV in Vero 76 cells, with the exception of YFV-17D, which replicated better in MEF-WT (Figure 3a–d). Loss of αVβ3 integrin expression in the MEF-αVβ3^−/−^ did not confer resistance to flavivirus infection. However, replication efficiency was significantly impaired in the MEF-αVβ3^−/−^ cells in comparison with the MEF-WT cells (Figure 3a–d). For the YFV-17D, no infectious virus was detected after the first 24 h in the MEF-αVβ3^−/−^. Ninety-six hours post infection, the MEF-αVβ3^−/−^ infected with YFV-17D displayed the lowest titers (3.0 TCID_50_/_mL_), followed by LGTV (3.25 TCID_50_/_mL_), WNV (3.75 TCID_50_/_mL_) and USUV (5.0 TCID_50_/_mL_). These results indicate that αVβ3 integrin is not required as a flavivirus attachment factor or entry receptor, but its expression significantly affects flavivirus replication efficiency in MEFs.

### 3.4. Absence of αVβ3 Integrin Expression Affects Internalization of Some but Not All Flaviviruses

**Figure 4:**
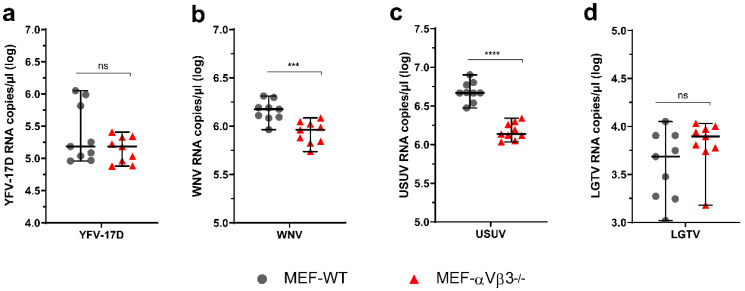
Internalization of (**a**) YFV-17D, (**b**) WNV, (**c**) USUV and (**d**) LGTV by MEF-WT and MEF-αVβ3^−/−^. Cells were inoculated with different flaviviruses on ice for 1 h to allow virus binding but not internalization. Thereafter, cells were extensively washed and shifted to 37 °C for 30 min to allow virus internalization. Cell monolayers were treated with acidic glycine (pH 2.5) for 2 min to inactivate non-internalized virus. Monolayers were harvested, the amount of internalized virus was determined by RT-qPCR and expressed as copy numbers per microliter (log transformed). Three independent experiments were performed in triplicate each. Dot plots represent each individual replicate from the three independent experiments. Statistical analysis: Unpaired Student’s *t*-test with Welch’s correction; (***) *p* ≤ 0.001; (****) *p* ≤ 0.0001; ns: not significant (*p* > 0.05).

Integrins promote a close connection with the actin cytoskeleton leading to a sequence of intracellular events that later culminate in remodeling of actin filaments and consequently cell membrane reorganization [48,49]. Flavivirus internalization is mediated mainly by clathrin-mediated endocytosis [7]. Since clathrin-mediated endocytosis is the main flavivirus entry and internalization route [7], we analyzed whether loss of αVβ3 integrin expression could hinder flavivirus internalization. Cells were incubated on ice prior to virus inoculation to stop cell membrane movement and prevent virus internalization and then inoculated with different flaviviruses and incubated for one hour. After 1 h, monolayers were washed and shifted to 37 °C to allow virus internalization. Internalized virus particles were quantified by RT-qPCR. As shown in Figure 4, the lack of αVβ3 integrin affected the internalization of WNV (*p* ≤ 0.001, Figure 4b), and USUV (*p* ≤ 0.0001, Figure 4c), but did not have any effect on internalization of YFV-17D (*p* > 0.05, Figure 4a) and LGTV (*p* > 0.05, Figure 4d). Together, these results suggest that αVβ3 integrin is involved in the internalization process of some, but not all flaviviruses.

### 3.5. Lack of αVβ3 Integrin Substantially Impairs Flavivirus Replication

Next, we investigated whether deletion of αVβ3 integrin affects flavivirus replication. The MEF-αVβ3^−/−^ as well as MEF-WT were inoculated with different flaviviruses at a MOI of 10, and 48 h post-infection, supernatants and monolayers were separately harvested and viral RNA load and virus titers were quantified by RT-qPCR and TCID_50_ determination, respectively.

**Figure 5:**
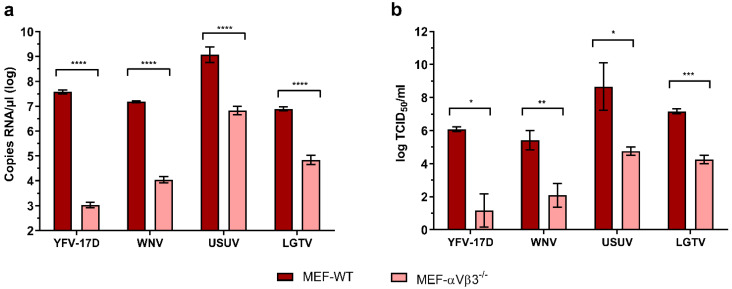
Replication analysis of YFV-17D, WNV, USUV and LGTV in MEF-WT and MEF-αVβ3^−/−^ cells 48 h post inoculation showing (**a**) viral RNA load and (**b**) virus titers. Cells were seeded into 12-well plates and inoculated with different flaviviruses at a MOI of 10. After one hour, monolayers were extensively washed and shifted to 37 °C for 48 h. Supernatants were harvested, viral RNA isolated and RT-qPCR was performed to determine viral RNA yields. The amount of virus genome is expressed as copy numbers per microliter (log transformed). End-point determination of virus titers were calculated using the Spearman–Kärber method and expressed as TCID_50_ log values. Three independent experiments were performed in triplicate each. Bars represent mean values and error bars represent the standard deviation (mean ± standard deviation). Statistical analysis: Unpaired Student’s *t*-test with Welch’s correction: (*) = *p* ≤ 0.05, (**) = *p* ≤ 0.01, (***) = *p* ≤ 0.001 and (****) = *p* ≤ 0.0001.

Interestingly, as shown in Figure 5, deletion of αVβ3 integrin significantly impaired replication of all flaviviruses investigated. In general, the reduction on viral RNA load in the MEF-αVβ3^−/−^ reached more than 90% for all four flaviviruses tested (Figure 5a) in comparison with the MEF-WT. Similarly, virus titers measured by TCID_50_ were drastically reduced in the MEF-αVβ3^−/−^ in comparison with the MEF-WT (Figure 5b). These results demonstrated that the absence of αVβ3 integrin expression strongly impaired flavivirus replication in MEFs. To confirm our findings, CHO cells expressing either the mouse αV or β3 integrin subunit were infected with four flaviviruses and viral RNA load was measured by RT-qPCR. As observed in Figure S5a–d, ectopic expression of αV integrin subunit in CHO cells (CHO-αV^+/+^) enhanced replication of YFV-17D by 82% (*p* = 0.0045) and USUV by 142% (*p* ≤ 0.0001) in comparison to CHO-K1 wild-type cells. Expression of β3 integrin subunit in CHO cells (CHO-β3^+/+^) had no effect on replication of YFV-17D and USUV but enhanced replication of WNV by 21.5% (*p* = 0.0251) and LGTV by 72.5% (*p* = 0.0069) in comparison to the CHO-K1 wild-type cells. Taken together, these results demonstrated that flavivirus replication benefits from the expression of integrins, either αV and/or β3 integrin subunit.

### 3.6. αVβ3 Integrin Knockout Influences Flavivirus RNA Genome Replication

**Figure 6:**
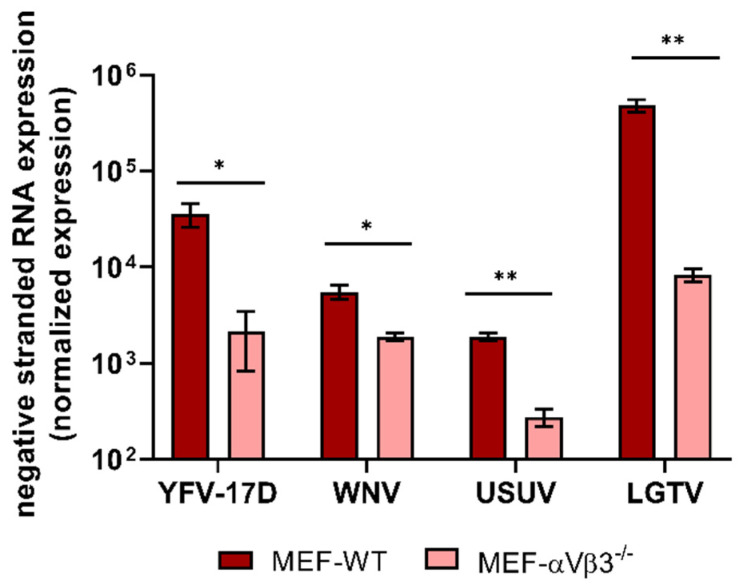
Relative quantification of flavivirus antigenome (negative-strand) RNA in MEF-WT and MEF-αVβ3^−/−^ cells. Cells were infected with YFV-17D, WNV, USUV and LGTV at a MOI of 10. After 48 h post inoculation, monolayers were washed, harvested, lysed and total RNA was extracted. RT-qPCR was performed to quantify the amount of negative-strand RNA. The levels of flavivirus negative-strand RNA were normalized against beta-actin, a housekeeping gene, and the relative gene expression was calculated by 2^^−ΔΔCt^ method. Levels of flavivirus negative-strand RNA were expressed as fold amplification in relation to the housekeeping gene. Three independent experiments were performed in triplicate each. Bars represent mean values and error bars represent the standard deviation (mean ± standard deviation). Statistical analysis: Unpaired Student’s *t*-test with Welch’s correction: (*) = *p* ≤ 0.05 and (**) = *p* ≤ 0.01.

Since flaviviruses are positive sense RNA viruses, a negative-strand anti-genomic viral RNA is synthetized during flavivirus RNA replication, which serves as a template for new positive stranded genomic viral RNA molecules. Detection of negative-strand anti-genomic viral RNA is thus considered a hallmark of active flavivirus replication [5,50]. The negative-stranded RNA is synthetized at low levels during the replication cycle. Some authors have proposed a ratio (positive/negative stranded) between 6:1 to 12:1 in the early stages of infection and 45:1 to 100:1 in the late stages of infection [51,52,53,54]. In order to confirm whether the lack of αVβ3 influences flavivirus replication at the RNA replication level, we measured the levels of flavivirus antigenomic RNA in MEF-WT and MEF-αVβ3^−/−^ cells. Interestingly, the levels of all four flavivirus antigenomic RNA were considerably lower in MEF-αVβ3^−/−^ than in MEF-WT (Figure 6). Synthesis of negative stranded RNA was reduced by 94% for YFV-17D, 65.7% for WNV, 85% for USUV and 98% for LGTV in the MEF-αVβ3^−/−^ when compared to the MEF-WT (100%). This finding strongly supports the involvement of αVβ3 integrin in flavivirus RNA replication.

## 4. Discussion

Although many aspects of flavivirus binding and entry into the host cells have been already described, cellular host factors modulating flavivirus replication are still an unexplored field. In the last few years, several new flavivirus host cell factors have been reported and the knowledge is increasing rapidly [55,56,57,58,59,60]. Integrins, one of the major families of cell adhesion molecules have been postulated to be involved in the flavivirus infection cycle [12,28,30,31,32]. In the present study, we demonstrated that αVβ3 integrin is not involved in flavivirus binding to the host cell, but indeed involved in flavivirus RNA replication and at some degree also in the internalization process of some flaviviruses.

MEFs lacking the expression of αVβ3 integrin were used in this study to investigate the involvement of this specific integrin in the flavivirus infection cycle. The double knockout MEF model used in the present study displays a full deletion of the αVβ3 integrin gene at the DNA level by homologous recombination similarly to what has been described elsewhere [32]. By targeting gene ablation at the DNA level, we avoided several off-target effects commonly observed in gene silencing techniques like siRNA/shRNA, which may partially influence the outcome of virus replication [61].

There are 24 different integrin molecules described and due to the nature of integrins to form heterodimers; deletion of one integrin subunit impairs the expression of other integrins at the cell membrane [12]. The MEF model used in our study displayed a full deletion in the αV and β3 integrin subunit genes, which, in turn, disables the expression at the cellular membrane of all αV and β3 integrin heterodimer combinations. Flow cytometry analysis revealed the total absence of αV and β3 integrin expression in the MEF-αVβ3^−/−^ cells. However, cells still expressed considerable levels of β1 integrin subunit, indicating that the ablation of either αV or β3 integrin subunits does not influence the expression of β1 integrin subunit. This integrin subunit may interact and form heterodimers with α integrin subunits other than αV. Several studies have demonstrated that MEFs express a diverse repertoire of integrins, in particular β1 integrin subunit combinations such as α5β1, α11β1, α2β1 and α1β1 [62,63,64,65,66]. Besides the αV and β3 integrin subunits investigated in our study, it remains unknown whether MEFs from our study express other integrin subunits beyond αV, β3 and β1. Additionally, it should be considered that the lack of one or more integrin subunits might lead to a compensatory effect in which cells upregulate the expression of other integrin heterodimers. Other studies demonstrated the susceptibility of MEFs to flavivirus infection leading to productive virus replication [29,32]. Several viruses, like foot and mouth disease virus, Epstein-Barr virus, echovirus and herpesviruses use integrins to mediate binding to the host cell [10]. In our study, we have shown that the loss of αVβ3 integrin expression did not confer cell resistance to any of the flaviviruses studied, and binding to αVβ3 expressing and non-expressing cells was similar, but replication efficiency was considerably impaired. Thus, the hypothesis that αVβ3 integrin might be a major flavivirus receptor or mediate flavivirus attachment to the cell membrane of MEF is not supported by our results. These findings also corroborated with previous reports demonstrating that the absence of integrin expression did not confer cell resistance to WNV and is not required for flavivirus binding to the host cell [29,32]. In the present study, CHO-K1 cells expressing the mouse αV or β3 integrin subunit were used as model to study the influence of integrin expression in flavivirus infection. Of note, CHO-K1 cells have been described to be resistant to a variety of viruses as several integrin subunits are not expressed [67]. In accordance with our results using MEFs lacking the expression of αVβ3 integrin, expression of either αV or β3 integrin subunit in CHO-K1 cells, *per se* did not increase flavivirus binding to the cell surface.

Chu et al. reported that antibodies against αV and β3 integrin subunits as well as integrin ligands inhibited WNV binding and internalization in CS-1 human melanoma cells [28]. Similarly, epitope-blocking antibodies targeting αV and β3 integrin subunits in baby hamster kidney (BHK) cells reduced JEV replication [30]. In our experiments, cell treatment with synthetic RGD tripeptide motif, vitronectin and type I collagen, all integrin ligands, did not affect flavivirus binding to MEFs so that the involvement of integrins in flavivirus binding is most unlikely. Thus, our results are in accordance with what has been reported previously for WNV [29,32]. In fact, several other attachment factors have been described for flaviviruses, among them C-lectin molecules, mannose receptors and heparan sulfate. Studies with YFV, DENV, TBEV and WNV could show that flavivirus pre-incubation with heparin or cell treatment with heparinase abrogated the infection at considerable amounts (more than 90%) [60,68,69,70,71,72,73]. Considering that the virus strains used in our study are attenuated or cell culture adapted strains, future studies should evaluate if pathogenic and/or not cell-culture adapted strains differ in their ability to bind heparan sulfate that could minimize flavivirus binding and usage of integrins.

Integrin-mediated intracellular signaling can promote cytoskeleton rearrangement and several viruses exploit this pathway for virus internalization [10,48,49]. Our results demonstrate that the absence of αVβ3 integrin expression affects internalization of WNV and USUV, but not that of YFV and LGTV. Since MEFs are likely to express integrin heterodimers other than αVβ3 integrin, one could speculate whether these viruses use different integrins for virus internalization. Additionally, the presence of other integrin heterodimers may be required for LGTV and YFV-17D internalization into MEFs. Further studies should investigate whether other integrin heterodimers may play a role in the internalization of other flaviviruses. The presence of integrin ligand motifs (RGD motif) in envelope proteins of YFV-17D, JEV and Murray Valley encephalitis virus was previously reported [74]. Amino acid exchanges in the YFV-17D RGD motif did not affect virus binding to or internalization into the target cell but rather had a negative effect on virus replication and spread [74]. Nevertheless, it might be possible that flaviviruses are dependent on integrins to promote internalization in a similar mechanism to what is described for hantaviruses and human cytomegalovirus (HCMV). Hantaviruses were demonstrated to bind to the plexin-semaphorin integrin domain of the inactive bent integrin conformation, while HCMV was demonstrated to bind integrins via a highly conserved disintegrin-like domain [24,75]. Further studies should address whether the integrin activated state might play a role in flavivirus infection.

Finally, we demonstrated that the deletion of αVβ3 integrin in MEFs markedly impaired replication of all flaviviruses tested here, leading to reductions in viral RNA loads, virus titers and decreased amounts of flavivirus antigenomic RNA. Moreover, ectopic expression of either αV or β3 integrin subunit in CHO cells slightly enhanced flavivirus replication. This evidence suggests that the αVβ3 integrin is a common flavivirus host cell factor, necessary for efficient flavivirus replication. Previous studies also showed that the expression of integrins in MEFs enhanced WNV replication in comparison to their β1 or β3 integrin-deficient cell line counterparts [32]. A second study with JEV showed that silencing the expression of αV or β3 integrin subunits in BHK and HeLa cells led to reductions on JEV replication rates reinforcing that integrins are a mutual flavivirus host cell factor. In the same study, expression of β3 integrin subunit in CHO cells enhanced JEV viral RNA loads and virus titers [30]. Interestingly, Chu et al., also demonstrated that rescue of β3 integrin subunit in CS-1 human melanoma cells greatly increased WNV entry and infectivity [28].

Some flaviviruses, as mentioned before YFV-17D, JEV and Murray Valley encephalitis virus, harbor the RGD motif in the envelope protein, suggesting that these flaviviruses might interact with integrins [74]. Whether the presence of this integrin ligand motif in some flaviviruses has any functional activity is unclear at present and should be further investigated. In accordance with van der Most et al., (1999) our experiments with YFV-17D showed the lowest replication rate in integrin αVβ3 deficient cells among all the other flaviviruses, reinforcing the importance of integrins for flavivirus replication [74].

Although the exact mechanism of how integrins modulate flavivirus replication is unclear, it might be conceivable that the integrin-mediated intracellular signaling is impaired once αVβ3 integrin is knocked-out. Therewith, other molecules that are under control of integrin expression or integrin-mediated intracellular signaling may be hampered in their function as important flavivirus host cell factors. For instance, integrins, in special αVβ3 integrin, have been shown to complex with Toll-like receptors (TLR) enhancing their responses to viral and bacterial agents. Depletion of TLR-2 and TLR-3 or β3 integrin in epithelial cells, keratinocytes and neural cell lines greatly impaired TLR responses culminating in less interferon and tumor necrosis factor responses. Further studies should investigate whether interferon and TLR signaling pathways are impaired once αVβ3 integrin is deleted in MEFs [76,77,78].

Integrins are very specialized molecules in transmitting and controlling several downstream pathways leading to diverse cellular responses such as cell migration, differentiation, mitoses and apoptosis [13]. A study conducted by Zaidel-Bar and colleagues demonstrated the magnitude of integrin interactions, called “integrin adhesome”, culminating in more than 150 cellular components and more than 690 interactions with diverse cellular components [79]. In this sense, it is plausible that the loss of αVβ3 integrin expression and consequently, impairment of its intracellular signaling pathways might interfere with the expression of several other molecules that might affect flavivirus RNA replication. Additionally, integrin-mediated intracellular signaling might enhance the expression of cellular molecules that promote flavivirus replication creating a more favorable environment for flavivirus replication.

In conclusion, our results strongly suggest that αVβ3 integrin is a mutual flavivirus host cell factor that influences the flavivirus replication efficiency. Further studies should be performed in mouse and human cell lines to dissect the exact mechanisms in which integrins modulate flavivirus replication.

## Data Availability

All data are given in the manuscript and the Supplementary Materials.

## Supplementary Materials

The following are available online at https://www.mdpi.com/article/10.3390/v14010018/s1, Table S1: Primers and probes used in this study; Figure S1: Etermination of cell viability and cell adhesion to vitronectin for mouse embryonic fi-broblast (MEF) wild-type (WT) and MEF-αVβ3^−/−^ cells; Figure S2: Integrin expression patterns in Chinese hamster ovary (CHO) cells; Figure S3: Flavivirus binding to the cell surface of Vero 76 cells; Figure S4: Binding inhibition assay in MEF-WT and MEF-αVβ3^−/−^; Figure S5: Flavivirus replication in Chinese hamster ovary (CHO-K1) cells expressing the αV or β3 integrin subunit. References [80,81,82] are cited in the supplementary materials.
