# Supplementary material for: αVβ3 Integrin Expression Is Essential for Replication of Mosquito and Tick-Borne Flaviviruses in Murine Fibroblast Cells
Source: Viruses. 2021 Dec 23;14(1):18. doi: 10.3390/v14010018 (PMC8780171; doi:10.3390/v14010018)
Supplement: Supplementary file 1 [file viruses-14-00018-s001.zip › viruses-1460101-supplementary.pdf]

Table S1. Primers and probes used in this study.

Target	Sequence (5'-3')	Ref
YFV-17D	YFV_Fw: TACAACATGATGGGAAAGAGAGAGAARAA<br>YFV_Rev: GTGTCCCAKCCRGCTGTGTCATC<br>Probe_YFV: FAM-TCAGAGACCTGGCTGCAATGGATGGT-TAMRA	[80]
WNV	WNV_Fw: TCAGCGATCTCTCCACCAAAG<br>WNV_Rev: GGGTCAGCACGTTTGTCATTG<br>Probe: FAM-TGCCCGACCATGGGAGAAGCTC-TAMRA	[81]
USUV	USUV_Fw: CGTTCTCGACTTTGACTA<br>USUV_Rev: GCTAGTAGTAGTTCTTATGGA<br>Probe_WNV: FAM-ACCGTCACAATCACTGAAGCAT-BHQ1	[34]
LGTV	LGTV_Fw: TACAACATGATGGGAAAGAGAGAGAARAA<br>LGTV_Rev: GTGTCCCAKCCRGCTGTGTCATC<br>Probe_LGTV: FAM-TGAAAAAACTGGCTTCCTTGAGTGGT-BHQ1	[80]
β-actin	ACTB_Fw: CAGCACAATGAAGATCAAGATCATC<br>ACTB_Rev: CGGACTCATCGTACTCCTGCTT<br>Probe_ACTB: VIC-TCGCTGTCCACCTTCCAGCAGATGT-TAMRA	[82]

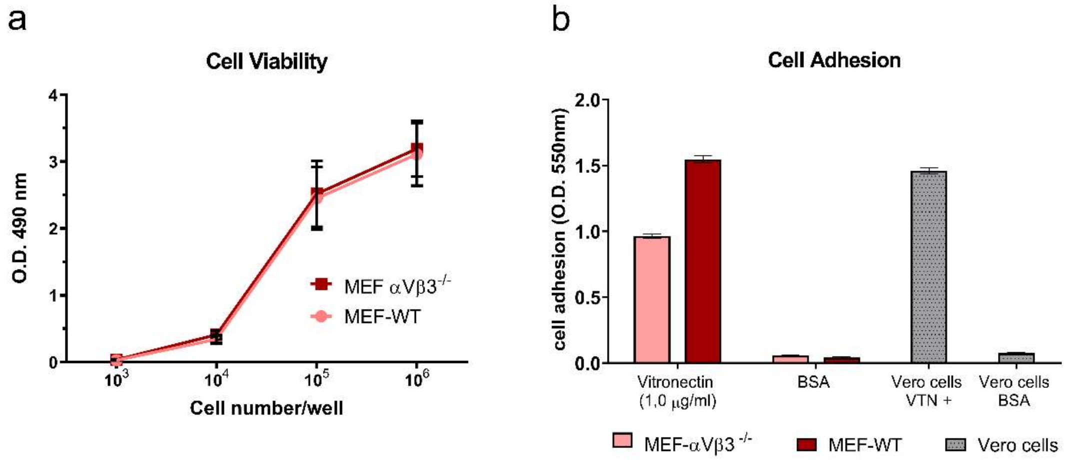

Figure S1. Determination of cell viability and cell adhesion to vitronectin for mouse embryonic fibroblast (MEF) wild-type (WT) and MEF-αVβ3$^{-/-}$ cells. **(a)** Cells were seeded at concentrations of 10^3 to 10^6 cells per well and incubated for 4 hours at 37° C with tetrazolium reagent. Optical densities were measured at 490 nm. Two independent experiments were performed each in duplicate. **(b)** Plates were coated with 1 μg/ml of recombinant mouse vitronectin (VTN) overnight. The day after, plates were blocked with 1% bovine serum albumin (BSA) and MEF-WT and MEF-αVβ3$^{-/-}$ cells were seeded and incubated at 37°C with 5% carbon dioxide (CO_2) for 30 minutes. Plates were washed to remove unbound cells. Attached cells were fixed with 3% paraformaldehyde (PFA) and stained with 1% crystal violet. Dye was extracted with citrate-ethanol buffer and optical density was measured at 550 nm. Vero 76 cells and BSA coated wells were used as controls for the assay. Two independent experiments were performed in triplicate each. Bars represent the mean values and error bars represent the standard deviation (means ± standard deviation).

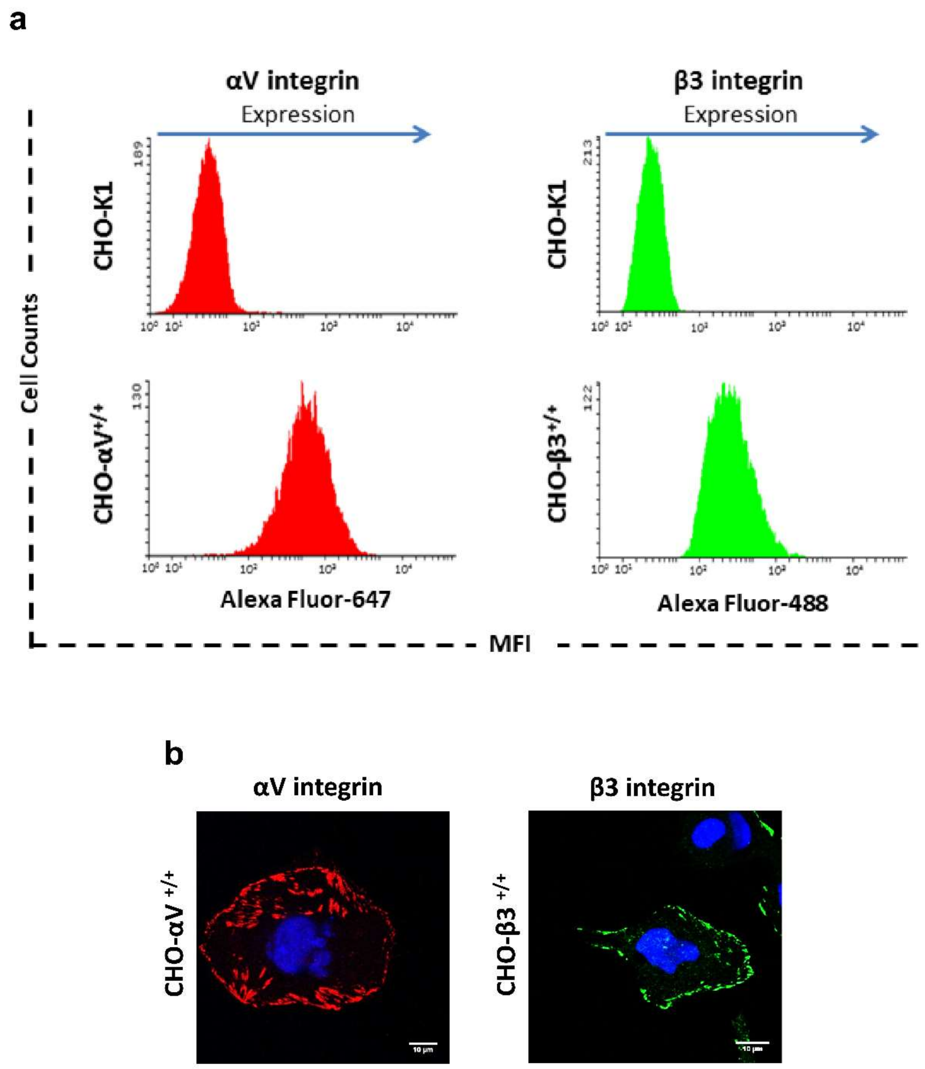

Figure S2. Integrin expression patterns in Chinese hamster ovary (CHO) cells. Cells were analyzed by flow cytometry **(a)** and laser scanning confocal microscopy **(b)**. In **(a)**, cells were incubated with anti-integrin subunit-specific antibodies followed by incubation with secondary antibodies labelled with Alexa 647 (red histograms) or Alexa 488 (green histograms). Cell controls correspond to untreated cells. Histograms represent the mean fluorescence intensity (MFI – x-axis) and the cell counts (y-axis). In **(b),** cells were cultivated on coverslips and stained with integrin subunit-specific antibodies followed by incubation with secondary antibodies labelled with Cy3 (red) or Alexa 488 (green). Nuclei were stained with 4',6-diamidino-2-phenylindole (DAPI). Scale bar: 10 μm.

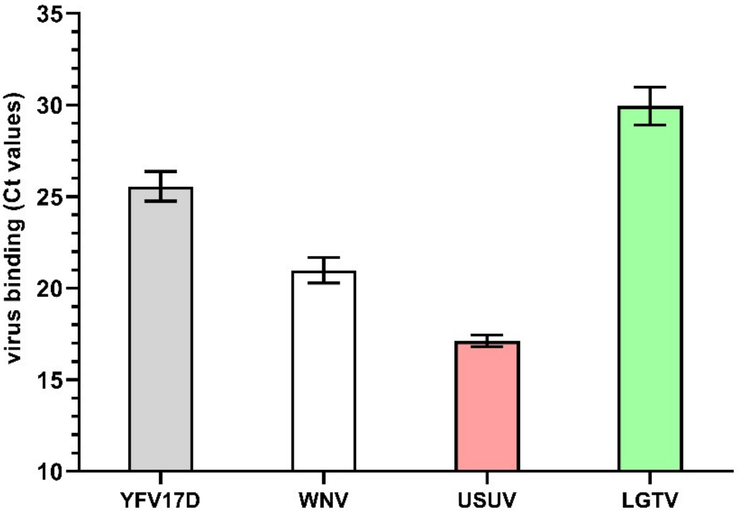

Figure S3. Flavivirus binding to the cell surface of Vero 76 cells. Cells were inoculated with yellow fever virus strain 17D (YFV-17D), West Nile virus (WNV), Usutu virus (USUV) or Langat virus (LGTV) at a MOI of 10 and placed on ice to allow virus binding to the cell surface but no internalization. After 1 hour, cells were extensively washed, monolayers harvested and the amount of virus bound to the cell surface was determined by RT-qPCR. Virus binding values are expressed in cycle threshold values (Ct values). Two independent experiments were performed in triplicate each. Bars represent the mean values and error bars represent the standard deviation (means ± standard deviation).

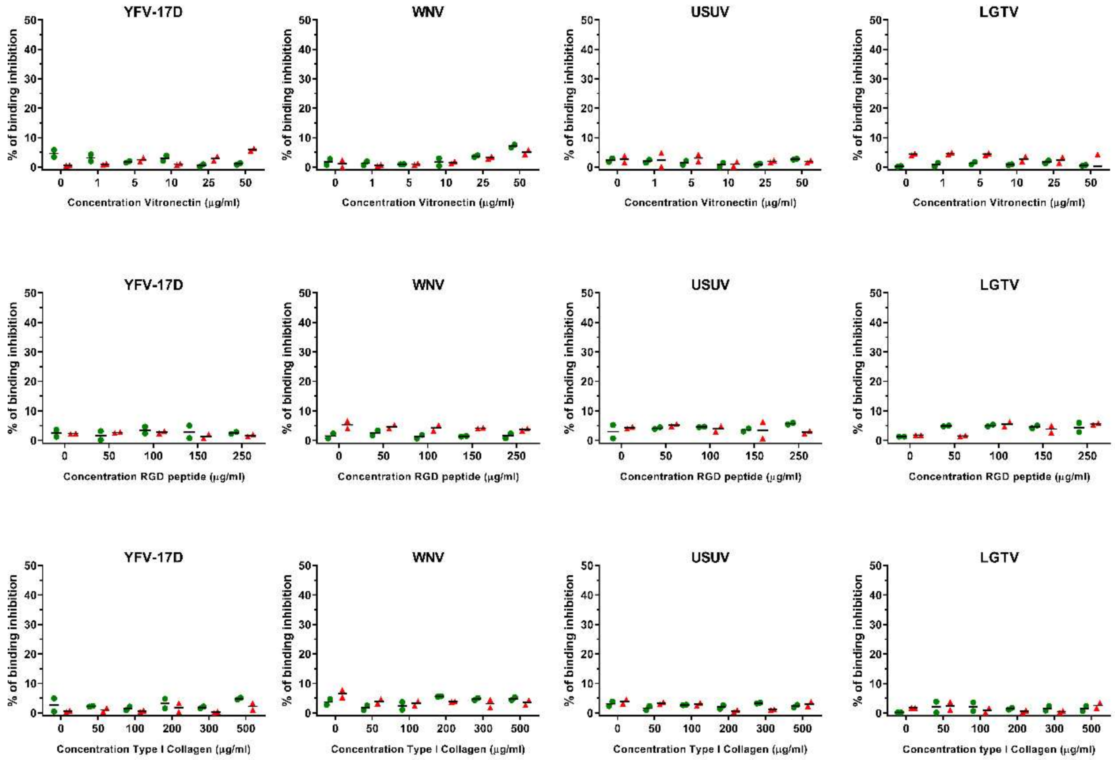

Figure S4. Binding inhibition assay with MEF-WT and MEF-$\alpha V\beta 3^{-/-}$. Cells were first treated with increasing concentrations of recombinant mouse vitronectin (0-50 µg/ml), synthetic cyclic arginine-glycine-aspartate (RGD) tripeptide (0-250 µg/ml) and type I collagen (0-500 µg/ml) for 30 minutes prior to virus inoculation. Thereafter, treated cells were shifted to 4°C and inoculated with

different flaviviruses at a multiplicity of infection (MOI) of 10 for 1 hour. After, monolayers were extensively washed and resuspended in RLT buffer. The total RNA was isolated and analyzed by RT-qPCR. Percentage of binding inhibition was calculated based on cycle threshold values. Two independent experiments were performed in duplicate each. Dots represent the mean of individual values from each independent experiment. Dashes represent the median.

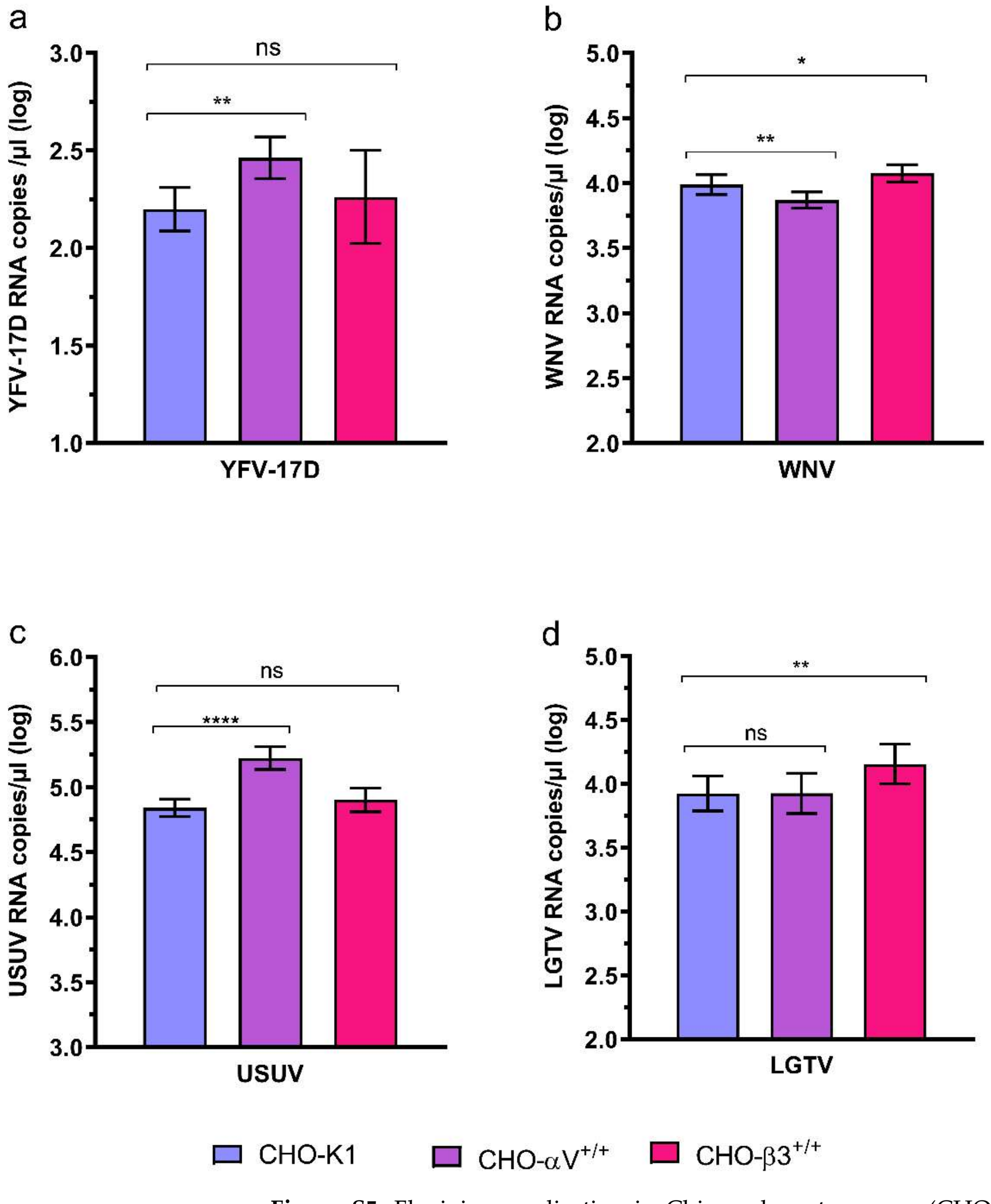

Figure S5. Flavivirus replication in Chinese hamster ovary (CHO-K1) cells expressing the αV or β3 integrin subunit. Cells were cultivated in 12-well plates and inoculated with YFV-17D 17D (**a**), WNV (**b**), USUV (**c**) and LGTV (**d**) at a MOI of 10 for 1 hour at 37°C. Thereafter, cells were washed and incubated for 48 hours at 37 °C. Supernatants were harvested at 48 hours post-infection and viral RNA yields were quantified by RT-qPCR. Bars represent the mean value and error bars represent the standard deviation. Three independent experiments were performed in triplicate each. Statistical analysis: One way ANOVA; *,$p \leq 0.05$; **,$p \leq 0.01$; ****,$p \leq 0.0001$ and ns: not significant ($p > 0.05$).

References

34. Jöst, H.; Bialonski, A.; Maus, D.; Sambri, V.; Eiden, M.; Groschup, M.H.; Günther, S.; Becker, N.; Schmidt-Chanasit, J. Isolation of Usutu Virus in Germany. *Am J Trop Med Hyg* **2011**, *85*, 551–553, doi:10.4269/ajtmh.2011.11-0248.

80. Vina-Rodriguez, A.; Sachse, K.; Ziegler, U.; Chaintoutis, S.C.; Keller, M.; Groschup, M.H.; Eiden, M. A Novel Pan-Flavivirus Detection and Identification Assay Based on RT-qPCR and Microarray. *Biomed Res Int* **2017**, *2017*, doi:10.1155/2017/4248756.
81. Lanciotti, R.S.; Kerst, A.J.; Nasci, R.S.; Godsey, M.S.; Mitchell, C.J.; Savage, H.M.; Komar, N.; Panella, N.A.; Allen, B.C.; Volpe, K.E.; et al. Rapid detection of west nile virus from human clinical specimens, field-collected mosquitoes, and avian samples by a TaqMan reverse transcriptase-PCR assay. *J. Clin. Microbiol.* **2000**, *38*, 4066–4071, doi:10.1128/JCM.38.11.4066-4071.2000.
82. Toussaint, J.F.; Sailleau, C.; Breard, E.; Zientara, S.; De Clercq, K. Bluetongue virus detection by two real-time RT-qPCRs targeting two different genomic segments. *J. Virol. Methods* **2007**, *140*, 115–123, doi:10.1016/j.jviromet.2006.11.007.
